# Organisation of care for patients using direct oral anticoagulants

**DOI:** 10.1007/s12471-020-01436-8

**Published:** 2020-06-08

**Authors:** A. J. W. Gulpen, J. K. van Dijk, N. L. Damen, H. ten Cate, S. Schalla, A. J. ten Cate-Hoek

**Affiliations:** 1 Thrombosis Expertise Centre Maastricht, Maastricht University Medical Centre, Maastricht, The Netherlands; 2 Cardiovascular Research Institute Maastricht (CARIM), Maastricht, The Netherlands; 3 Department of Cardiology, Maastricht University Medical Centre, Maastricht, The Netherlands; 4 Department of Internal Medicine, Maastricht University Medical Centre, Maastricht, The Netherlands; 5 Ardis, The Hague, The Netherlands; 6 Elisabeth-TweeSteden Hospital, Tilburg, The Netherlands

**Keywords:** Direct oral anticoagulation, Atrial fibrillation, Functional resonance analysis method, Anticoagulation care

## Abstract

Direct oral anticoagulants (DOACs) are recommended by several scientific societies as first-line therapy for the prevention of stroke and systemic embolism in patients with atrial fibrillation. However, there is uncertainty regarding the organisation of anticoagulation care, with various caregivers being involved. Patients and caregivers are often confronted by uncertainty about the coordination of treatment. With the functional resonance analysis method we visualised the process of anticoagulation care in daily practice in the Maastricht region. This resulted in recommendations on how to improve the organisation of anticoagulation care for DOAC patients.

## Current situation regarding anticoagulation care in the Netherlands

Anticoagulation therapy is effective for patients with an increased risk of thromboembolic events. Direct oral anticoagulants (DOACs) are replacing vitamin K antagonists (VKAs), primarily driven by new guidelines, in which DOACs are preferred to VKAs [1–11]. This change has major consequences for the anticoagulant care landscape. In the Netherlands, VKA patients are actively monitored and supervised by the anticoagulation clinic (AC). Patients that start on a DOAC are no longer under regular surveillance by the AC, as their service is exclusively funded by insurance companies, based on INR performance and dosing advice, linked to VKAs. Monitoring of DOAC patients was initially predominantly provided by medical specialists, nowadays increasingly by general practitioners (GPs). Optimal anticoagulant treatment should preferably involve personalised medicine, which in an era of switching from VKAs to DOACs will take time to be implemented [11, 12]. The Dutch National Standard for Integrated Care (LSKA 2.0) states that care must be organised for all patients on (any form of) antithrombotic medication. One major issue in care for DOAC patients is the lack of a structured follow-up. The LSKA 2.0 recommends an at least annual evaluation of all aspects of anticoagulation, including laboratory tests for liver and kidney functions. In addition, adherence to medication should be verified, and complications and side-effects must be recorded, as recommended by national (LTA 2019, in preparation) and international guidelines [11, 13]. All these aspects are clinically relevant, as both non-adherence and prescription of too low doses of DOAC are frequently observed in real-life studies [14, 15]. The upcoming Dutch guideline (LTA 2019) is in agreement with this, and states that the responsibility in DOAC treatment and follow-up should be shared between patient, pharmacist and medical specialist.

## Analysing the current situation with the functional resonance analysis method

Many causes of failure to comply with guidelines and of lack of adherence to medication have been considered [16]. In addition to the search for guideline non-adherence and root causes of complications (the Safety‑I approach), a promising new perspective has become available, with insight into what can be learned from daily practice, providing the basis for improvement initiatives [17]. This Safety-II perspective tries to understand how processes usually go right and how this relates to predefined procedures, such as protocols [18]. A useful tool for this purpose is the functional resonance analysis method (FRAM), which has been endorsed by safety experts, as a promising way to improve safety in complex systems such as healthcare [19].

FRAM is a method to visualise processes as they are carried out in everyday practice (‘work-as-done’). Based on this information, good practices in the process can be identified, but also points for improvement and possible risks. As described previously [20, 21], the findings of a FRAM analysis help to initiate practically feasible improvement initiatives. This may contribute to quality and safety management, optimisation of work processes, prospective risk analysis, analysis of incidents and development and implementation of protocols [19, 22, 23].

**Fig. 1:**
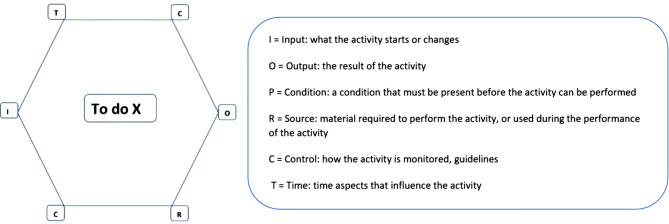
The functional resonance analysis method (FRAM) function with all aspects. (To do X can represent any activity). *I*  Input: what the activity starts or changes, *O* Output: the result of the activity, *P* Condition: a condition that must be present before the activity can be performed, *R* Source: material required to perform the activity, or used during the performance of the activity, *C* Control: how the activity is monitored, guidelines, *T* Time: time aspects that influence the activity

All essential activities that build up a process are described in models. In these models, activities are depicted as hexagons with six different labels (Fig. 1). Information on these activities can be obtained from various sources, including interviews and documentation. For detailed information on FRAM, we refer to practical instruction guides and previous publications [19–23].

**Fig. 2:**
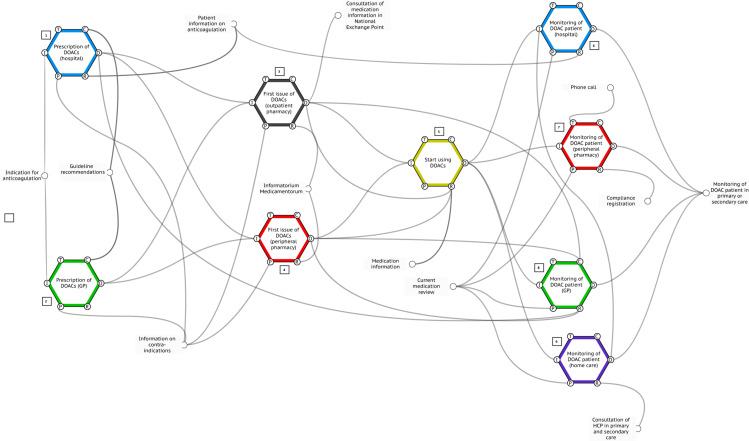
Work-as-done FRAM model of the organisation of care for patients using direct oral anticoagulants (DOACs), beginning in the *left* part of the figure with prescription of a DOAC by a medical specialist or GP (*1*, *2*). Then the patient goes to the pharmacy to pick up the DOAC (*3*, *4*). Thereafter, the patient starts taking the DOAC (*5*), and monitoring is done by various healthcare providers in primary or secondary care (*6*–*9*). (*HCP* healthcare professionals)

We used FRAM to gain insight into how care for patients on DOAC is arranged in daily practice, from the moment of prescription in the hospital setting, up to and including the follow-up (Fig. 2). This was based on semi-structured interviews with the following stakeholders: general practitioners (2), home care nurse (1), coordinator of home care for patients with ischaemic cerebral events (1), dentist (1), pharmacists (2), cardiologists (2), gastroenterologist (1), neurologist (1), internist (1). We questioned them about their role in the process and their collaboration with other professionals. One patient was also interviewed.

After written consent, interviews were audio recorded and summarised immediately afterwards. Interviews were guided by a topic list, with minor adjustments made per specific discipline [21] and were conducted until data saturation was reached [24]. Afterwards, a discussion meeting was organised to present the final model to the stakeholders and other authors as a means of validation, and to elaborate on potential clinical implications and recommendations. The analysis was done under the auspices of the Thrombosis Committee of the Maastricht University Medical Centre.

We identified the most important points for improvement in the process and structured the findings of our FRAM analysis based on four perspectives: task division and role clarity, multidisciplinary collaboration, efficiency, and guidance and support [23].

### Task division and role clarity

Our study showed that there is no agreement on follow-up responsibilities, with both GPs and medical specialists not knowing who is responsible. Primary care stakeholders indicated that for them it is often unclear which medical specialist is responsible. This is due to the fact that the main responsibility is dependent on the situation rather than structural. For example, if a patient is treated by a cardiologist, renal function is checked during the periodic visit. However, the frequency of visits differs per patient. The internist pointed out that even if renal function is checked, no action is taken when changes in clearance are observed. Because of this lack in clarity about the role that each professional has within the anticoagulation chain, assumptions are made about each other’s role.

### Multidisciplinary collaboration

Multidisciplinary collaboration between peripheral pharmacy and GPs is good because their electronic systems are linked. However, the way information between transmural pharmacy, GP and medical specialists is shared, is less structured. The national database (*Landelijk Schakel Punt*, LSP) used by the pharmacy is hardly used by GPs or medical specialists. Apart from the LSP, there is no standard communication method between medical specialists and GPs. In particular, the transfer of renal function monitoring is highly variable. The letter of discharge from the hospital or outpatient clinic sent to the GP sometimes includes the advice to check renal function, but this information is often lacking. There is also no check on whether this advice has been followed by the GP. Furthermore, structural transmission of information to home care nurses is lacking. Especially the actual medication overview (AMO), a document that lists all medication that is currently taken by the patient, is often missing or incomplete. Medical specialists indicated that, overall, they assume that the pharmacy ‘will know’ about the medication. However, home care nurses are dependent on the AMO to be able to administer the correct medication to the patient.

### Efficiency

If the AMO is missing, it takes home care a lot of extra time to retrieve the data from the doctor/pharmacy or the patient. Although there is a policy regarding the provision of AMOs, adherence to this policy is still poor.

### Guidance and support

Overall, the information at the start of DOAC treatment is adequate. Most professionals prefer DOACs, mainly for convenience reasons, but according to their perspective this could also be a pitfall. Healthcare providers are not always aware of the risks of the DOAC. Also, the lack of routine as well as knowledge gaps might have an impact. Furthermore, the convenience aspect may lead to incautious behaviour on the part of patients.

The ‘peripheral’ pharmacy has some insight into therapy compliance; their system provides a signal when patients should come to collect their follow-up DOAC prescription. This means that the pharmacy checks compliance at least once every 3 months. This is in contrast to the in-hospital pharmacy, which has very limited insight into therapy compliance. Although it is mandatory to consider renal function when prescribing a DOAC, this is not always done or checked. At the pharmacy, renal function is considered when filling the first prescription but not for repeat prescriptions.

## How to improve anticoagulation care

This FRAM analysis provided insight into the process of care for DOAC patients in daily practice. Based on this analysis, we recommend the following to improve the organisation of anticoagulation care for DOAC patients.

First of all, it is necessary to clarify roles and responsibilities for medical specialists involved in the monitoring of DOAC patients. Who does what and how frequently? It is important that everyone is aware of these agreements, so that no incorrect assumptions are made. Therapy compliance and dosage are currently not structurally monitored and should be incorporated in patient follow-up. It will be helpful to structure follow-up, for example with automated signals, doctor alerts for renal function or, if needed, by organising care within a specialised DOAC clinical care pathway or clinic.

Further, the information transfer between professionals needs to be optimised. It is desirable that transfer takes place in a more timely, consistent, accurate and systematic way. This requires clear agreements and adequate action by the professionals involved. Ideally, using a single, generally accessible information system would greatly improve information transfer.

It is also recommended that knowledge about DOACs be increased among professionals. Not every professional is aware of the potential risks of DOACs; in particular, primary care professionals (home care, GP and dentist) lack knowledge and routine.

## Limitations

This FRAM analysis was performed in the Maastricht area. It can be expected that a FRAM analysis performed in other regions of the Netherlands might have differed to some extent. However, knowledge of internal and external variability was considered when the model was analysed. Knowledge of how care for patients using DOACs is organised in different regions of the Netherlands led us to conclude that this FRAM analysis is representative of the overall situation in the Netherlands.
